# Prospective evaluation of oxygen saturation endoscopic imaging for radiotherapy response in head and neck cancer

**DOI:** 10.1038/s43856-026-01752-x

**Published:** 2026-07-01

**Authors:** Kento Tomizawa, Atsushi Motegi, Hidenari Hirata, Hiroki Yamashita, Hironori Sunakawa, Yusuke Yoda, Wataru Okano, Tomohiro Enokida, Takeshi Fujisawa, Masaki Nakamura, Hidehiro Hojo, Seiichiro Abe, Madoka Sakuramachi, Hiroshi Igaki, Yutaka Saito, Shingo Sakashita, Kazuto Matsuura, Makoto Tahara, Tomonori Yano, Sadamoto Zenda, Tetsuo Akimoto

**Affiliations:** 1 Department of Radiation Oncology, National Cancer Center Hospital East, Kashiwa, Japan; 2 Gunma University Heavy Ion Medical Center, Maebashi, Japan; 3 Division of Radiation Oncology and Particle Therapy, Exploratory Oncology Research and Clinical Trial Center, National Cancer Center, Kashiwa, Japan; 4 Department of Gastroenterology and Endoscopy, National Cancer Center Hospital East, Kashiwa, Japan; 5 Division of Endoscopy, Saitama Cancer Center, Saitama, Japan; 6 Department of Head and Neck Surgery, National Cancer Center Hospital East, Kashiwa, Japan; 7 Department of Head and Neck Medical Oncology, National Cancer Center Hospital East, Kashiwa, Japan; 8 Department of Radiation Oncology, University of Tsukuba, Tsukuba, Japan; 9 Endoscopy Division, National Cancer Center Hospital, Tokyo, Japan; 10 Department of Radiation Oncology, National Cancer Center Hospital, Tokyo, Japan; 11 Division of Pathology, Exploratory Oncology Research & Clinical Trial Center, National Cancer Center, Kashiwa, Japan; 12 Department of Radiation Oncology, The Jikei University School of Medicine, Tokyo, Japan

**Keywords:** Tumour biomarkers, Head and neck cancer, Cancer imaging

## Abstract

**Background:**

We developed an endoscopic imaging technology, oxygen saturation imaging (OS imaging), to assess real-time tissue oxygenation levels. However, its clinical impact remains unclear. This prospective observational study evaluated the association between pretreatment tumor oxygen saturation (StO_2_) quantified by OS imaging and the outcomes of patients with head and neck squamous cell carcinoma (HNSCC) receiving radiotherapy (RT).

**Methods:**

The primary endpoint was the association between pretreatment tumor StO_2_ and complete response rate at 8 weeks after RT completion. Secondary endpoints were the impact of StO_2_ on 1-year overall survival, progression-free survival, and local failure rates.

**Results:**

Of 50 patients with HNSCC enrolled from 2019 to 2022, 42 who underwent RT and OS imaging are analyzed. Of those, 41 received concurrent platinum-based chemotherapy. Median pretreatment StO_2_ is significantly lower in tumor tissue than in normal mucosa (61% vs. 66%, respectively). Complete response rates at 8 weeks after RT are not significantly different between the hypoxia (StO_2_ ≤ 61%, n = 22) and non-hypoxia (StO_2_ > 61%, n = 20) groups (86% vs. 100%, respectively). After a median follow-up of 36 (range: 9–53) months, the hypoxia group has significantly worse 1-year overall survival (91% vs. 100%), progression-free survival (77% vs. 95%), and local failure (18% vs. 5%) rates than the non-hypoxia group.

**Conclusions:**

Despite not meeting the primary endpoint, our findings suggest that pretreatment tumor StO_2_ is correlated with 1-year outcomes after RT, and OS imaging may be useful for identifying RT-resistant hypoxic tumors in HNSCC.

## Plain language summary

Parts of cancers often have limited access to oxygen (hypoxia) due to the tumor growing so large there is an insufficient blood supply to all parts of the tumor. Hypoxia can result in radiotherapy being less effective at treating tumors and is associated with poor prognosis in a type of cancer called head and neck squamous cell carcinoma (HNSCC). Although various methods have been developed to detect hypoxia within a tumor, none are used clinically. We developed a minimally invasive imaging technology using a flexible tube with a camera that can assess the amount of oxygen within a tumor repeatably and in real-time. We found that finding a low level of oxygen within a tumor by our technology was associated with worse clinical outcomes in patients with HNSCC receiving radiotherapy. Our method may be useful for identifying hypoxic tumors with poor outcomes after radiotherapy. It may also potentially help guide therapeutic decisions for patients with HNSCC.

## Abstract (web summary)

Tomizawa et al. prospectively evaluate tumor oxygen saturation in head and neck cancer before radiotherapy using a functional endoscopic imaging technology that they developed. Lower tumor oxygen saturation is associated with worse 1-year outcomes, suggesting potential utility for identifying radioresistant hypoxic tumors.

## Introduction

Head and neck squamous cell carcinoma (HNSCC) is the seventh most common cancer worldwide, and its incidence is estimated to increase by 30% by 2030^1^. Radiotherapy (RT) plays a pivotal role in the management of HNSCC, providing effective tumor control and functional preservation^2^. However, hypoxia causes radioresistance and is associated with a poor prognosis in HNSCC^3–6^. HNSCC exhibits substantial heterogeneity in tumor hypoxia levels across patients^7^. Therefore, it is essential to establish a method for identifying hypoxic tumors in patients with HNSCC receiving RT.

Various methods have been developed to measure tumor hypoxia including direct oxygen measurements, imaging, immunohistochemistry, and gene/protein expression analysis^8^. Despite these developments, no method has been established in routine clinical practice. Conventional approaches are invasive, difficult for daily use, or not entirely accurate in detecting tumor hypoxia. Although polarographic needle electrodes can directly measure tumor hypoxia, they are invasive and susceptible to variation depending on the sensor’s location^9^. Imaging technologies for detecting tumor hypoxia, such as ^18^F-fluoromisonidazole-positron emission tomography, have the advantage of being less invasive. However, their resolution is low, and those based on blood perfusion are indirect and thus less specific^10^. Evaluation of tumor hypoxia using immunohistochemistry and gene/protein expression analysis is not real-time or easily repeatable. There is a need for alternative technologies, especially less invasive, real-time, and repeatable estimates of tumor hypoxia.

We have collaboratively developed an endoscopic imaging technology, oxygen saturation imaging (OS imaging; FUJIFILM, Tokyo, Japan), to assess real-time tissue oxygen saturation (StO_2_^11–13^). OS imaging can obtain conventional endoscopic images and pseudocolor maps of StO_2_ without the need for additional invasive procedures. Furthermore, imaging can be repeated during the treatment and follow-up periods. OS imaging is a functional endoscopic technique that can acquire real-time imaging of oxygen saturation in tumor and surrounding tissues by detecting the spectral differences between oxy- and deoxyhemoglobin. The robustness of OS imaging technology has been validated using blood vessel phantoms, in vivo tumor imaging in animal models, and human tumor tissues. However, the associations between the OS imaging findings, such as pretreatment StO_2_, and clinical outcomes have not been investigated. Therefore, the clinical utility of OS imaging in patients with cancer, including HNSCC, remains unclear.

Here, we report the results of a prospective observational study to evaluate the association between pretreatment tumor StO_2_ quantified by OS imaging and the clinical outcomes of patients with HNSCC receiving RT, predominantly with concurrent chemotherapy. Our findings suggest that lower pretreatment tumor StO₂ is associated with poorer outcomes after RT, supporting the potential utility of OS imaging for identifying RT-resistant hypoxic tumors in HNSCC.

## Methods

### Patient selection

This prospective observational study was conducted at two institutions. We enrolled patients who fulfilled the following criteria: (1) histologically confirmed oropharyngeal or hypopharyngeal squamous cell carcinoma or laryngeal squamous cell carcinoma with involvement of the oropharynx or hypopharynx, (2) ability to measure tumor StO_2_ before treatment using OS imaging, (3) indication for definitive RT (clinical stage I–IVB HNSCC [stage I–III only for p16-positive oropharyngeal squamous cell carcinoma] based on the 8th edition of the UICC TNM classification), (4) age ≥ 20 years, and (5) written consent obtained for undergoing OS imaging. We excluded patients in whom tumor StO_2_ was potentially difficult to assess using OS imaging quantitatively. This study was approved by the institutional ethical review committee of National Cancer Center Hospital East (approval number: 2018-275) and was conducted in accordance with the guidelines for biomedical research specified in the Helsinki Declaration of 1975 (as revised in 1983). Written informed consent was obtained from all patients before enrollment.

### Measurement of tissue StO_2_ by OS imaging

The method of quantifying StO_2_ in tissues using OS imaging has been reported previously^11^. The principle of StO_2_ measurement using OS imaging is to derive the variation in absorption spectra between oxy- and deoxyhemoglobin using a narrow band at 473 nm during endoscopy. Also, the green band (500–600 nm) in the visible spectrum is used to correct the hematocrit level influencing the reflectance of tissue. The red band (600–700 nm) is used to detect the change in illumination caused by the distance between the light exit window of the endoscope and the observed tissue. We synchronously captured conventional white-light endoscopic images and OS imaging using a pseudocolor map of the tissue StO_2_.

To determine StO_2_ levels in the pretreatment tumor tissue and adjacent normal mucosa of each patient, still images captured simultaneously during endoscopy, both white-light endoscopic images and pseudocolor maps of StO_2_, were utilized. The entire tumor was drawn on white-light endoscopic images by two endoscopists. Both endoscopists have Japan Gastroenterological Endoscopy Society certification and 9 years of clinical experience. The oxygen saturation image was overlaid on the white-light endoscopic image, and the StO_2_ value of the tumor area with ~1845 pixels was extracted using dedicated software. The region of interest in normal mucosa was designated as the entire tissue area excluding the tumor lesion. We calculated the median StO_2_ values of the tumor tissue and normal mucosa for each patient as described previously^11,13^; these values were used in subsequent analyses. StO_2_ levels obtained by OS imaging were not disclosed to the treating physicians and were used independently for research purposes.

### RT and chemotherapy

**Fig. 1:**
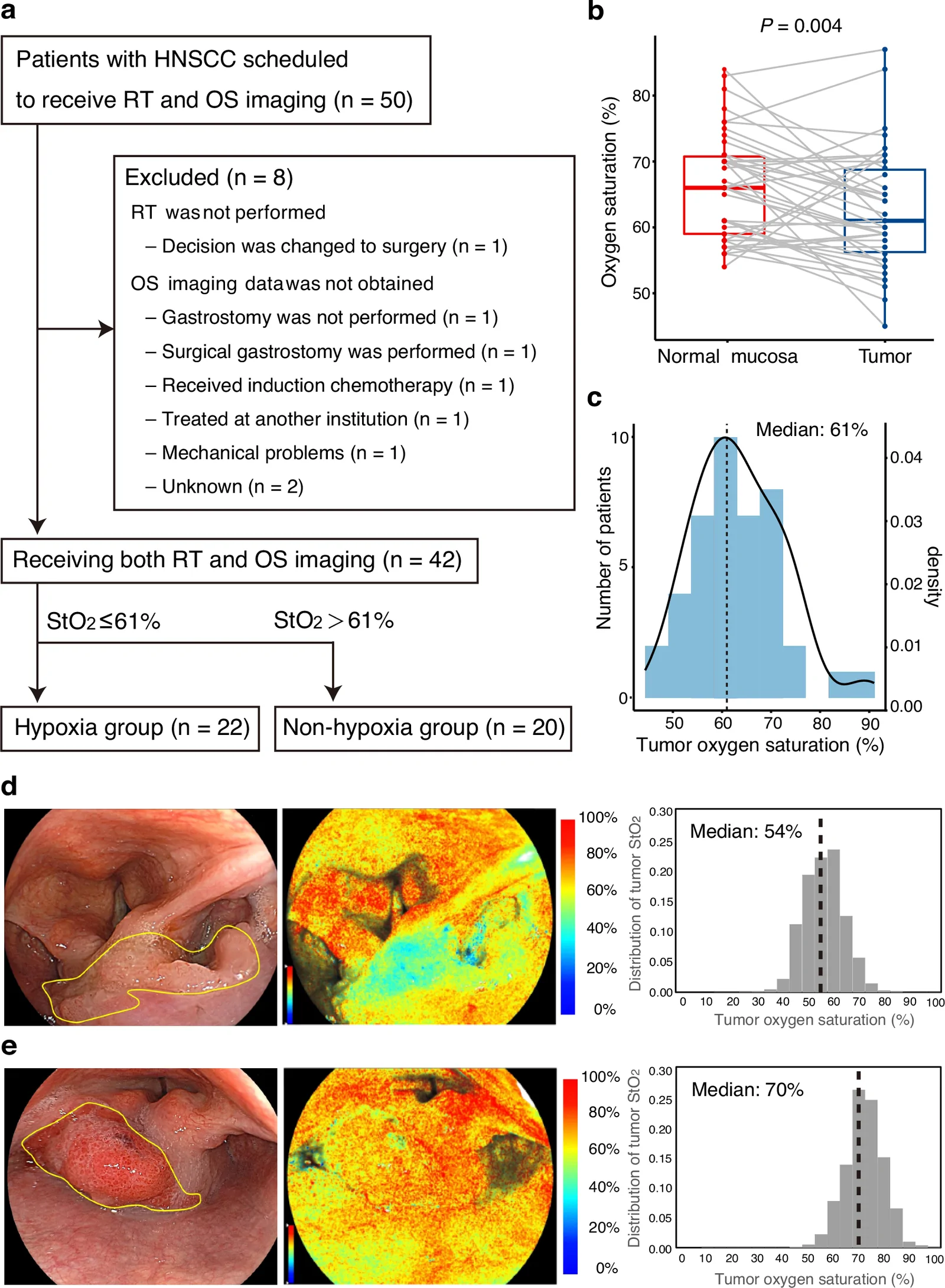
Study schematic and oxygen saturation imaging (OS imaging). **a** CONSORT diagram, showing 50 patients enrolled and 42 included in the analysis. **b** StO_2_ levels in pretreatment tumor tissue (*n* = 42) versus normal mucosa (*n* = 42), with both types of tissue samples obtained from each of the 42 patients (two-sided Wilcoxon signed-rank test). **c** Histogram showing the number of patients with each pretreatment tumor StO_2_ level among the 42 evaluated patients. **d**,**e** Representative images and distribution of tissue oxygen saturation (StO_2_) levels in hypoxic (**d**) and non-hypoxic (**e**) tumors measured by OS imaging. White light images were obtained by endoscopic observation. Segmentations of each tumor region are shown (left). Each StO_2_ map was visualized using OS imaging (middle). The histograms show the distribution of tumor StO_2_ in each patient (right).

A CONSORT diagram is shown in Fig. 1a. OS imaging was performed at the time of endoscopy for gastrostomy before RT. RT was administered via intensity-modulated radiation therapy and consisted of 35 fractions delivering 70 Gy to the macroscopic tumor, 63 Gy to the subclinical volume including high-risk nodal regions, and 56 Gy to elective regions. Platinum-based chemotherapy was concurrently combined with RT. Patients received cisplatin at either 80–100 mg/m^2^ once every 3 weeks or 40 mg/m^2^ weekly in up to seven administrations. When the physician deemed it difficult to administer the above drugs due to a poor general condition or severe complications, the drugs were omitted or reduced in dose. After RT completion, follow-up visits were conducted every 2–3 months during the first year, every 4–5 months during the second and third years, and every 6 months thereafter. Each follow-up visit included physical and laryngoscopic examinations. Post-treatment computed tomography (CT) was performed at 2–3 months after RT completion and every 6 months thereafter.

### Outcomes

The primary endpoint was the association between pretreatment tumor StO_2_ and complete response (CR) rate of the primary tumor site at 8 weeks after completion of RT. The determination of a CR or non-CR at the primary tumor site was assessed by the investigator using laryngoscopic images. CR was defined as the disappearance of the primary tumor on laryngoscopic examination; because we evaluated the StO_2_ level of the primary tumor, assessment of the response of the involved lymph node was not included. The secondary endpoints were the impact of pretreatment tumor StO_2_ on 1-year overall survival, progression-free survival (PFS), and cumulative incidence of local failure (LF) rates at the primary tumor site where tumor StO_2_ was assessed. Overall survival was calculated from the date of RT initiation to the date of death from any cause. PFS was calculated from the date of RT initiation to the date of any progression, including locoregional or distant failure, or death from any cause. LF was defined as disease progression at the primary tumor site after completion of RT, as assessed by the investigator using laryngoscopy, CT, or magnetic resonance imaging. In patients who achieved CR, LF was defined as the appearance of new lesions at the primary tumor site. In patients without CR, LF was defined as unequivocal progression of the primary tumor requiring a change in therapy.

### Evaluation of HIF-1α expression

In an exploratory analysis, we assessed the relationship between tumor hypoxia, as measured by OS imaging, and expression of HIF-1α, a key molecular marker of hypoxia, in HNSCC pretreatment biopsy specimens. Immunohistochemical staining was performed using a primary rabbit polyclonal antibody against HIF-1α (GTX127309, GeneTex) diluted at 1:300 as previously described^13^. Tumor StO_2_ values were not disclosed to the pathologist who independently reviewed the immunostaining results. HIF-1α expression was considered positive when 10% or more of the nuclei were stained.

### RNA sequencing (RNA-seq) and expression-based hypoxia scoring

As an exploratory objective, we evaluated the relationship between tumor hypoxia, as assessed by OS imaging, and the gene expression-based hypoxia profile. We performed RNA-seq of four pretreatment tumor samples collected from two patients (two samples/patient). Endoscopic biopsy samples were stored in RNAlater (QIAGEN) and kept at −80 °C until RNA extraction. The library used for RNA-seq was prepared using the SMART-Seq v4 Ultra Low Input RNA Kit for Sequencing (Takara Bio) and Nextera XT DNA Library Preparation Kit (Illumina). Sequencing was conducted from both ends of each cluster using the NovaSeq 6000 (Illumina). FASTQ files were mapped to the human reference genome (GENCODE v39), and aligned reads were converted for counting using the DRAGEN Bio-IT Platform (v3.7.5).

Based on a previous study, the hypoxia score for each sample was calculated by gene set variation analysis based on HNSCC mRNA-based hypoxia signatures^14,15^. We performed gene set enrichment analysis (GSEA)^16^ using hypoxia scores as a phenotype label to evaluate whether the biological pathways, the hallmark gene sets (v2023.2), are associated with tumor hypoxia.

### Data acquisition of external datasets

We analyzed RNA-seq data from 560 patients with HNSCC in The Cancer Genome Atlas (TCGA) dataset (HNSC_RNASeqV2_final_geneExp, https://gdc.cancer.gov/about-data/publications/PanCan-Squamous-2018/, last accessed on February 21, 2024). For patients in the dataset, appropriate consent was obtained, as approved by the relevant Institutional Review Board (IRB)^17^. For the Cancer Cell Line Encyclopedia (CCLE) analysis, RNA-seq data from 60 HNSCC cell lines were obtained from the Cancer Dependency Map portal (Expression Public 23Q2, https://depmap.org/portal/, last accessed on November 8, 2023) as described previously^18^.

To validate the correlation between StO₂ levels and a hypoxia-related molecular marker, we analyzed a previously reported independent dataset comprising 32 esophageal specimens (16 superficial esophageal squamous cell carcinoma specimens and 16 non-neoplastic tissue specimens) for which StO₂ values obtained by OS imaging and HIF-1α expression levels were available^13^. The previous study was conducted with approval of the IRB of National Cancer Center Hospital East (approval number: 2020–361). The IRB also approved the use of data in this study.

### Statistical analysis

Differences in CR rates of the primary tumors at 8 weeks after RT completion between the subgroups were examined using Fisher’s exact test. The median follow-up period was calculated using the reverse Kaplan–Meier method. The overall survival and PFS rates were estimated using the Kaplan–Meier method, and differences in survival curves were examined using the log-rank test. The LF rate was estimated using the cumulative incidence function, and the competing risk of death without LF was compared using Gray’s test. We completed the follow-up on December 26, 2023. Continuous variables were presented as medians with ranges and compared using the Wilcoxon rank-sum test. Categorical variables were compared using Fisher’s exact test. The degree of linearity was estimated by Spearman’s rank correlation coefficient. The Shapiro–Wilk test was used to assess normality. StO_2_ levels in both the tumor and normal mucosa of each patient were compared using the Wilcoxon signed-rank test. A two-sided *P*-value < 0.05 was considered significant. In GSEA, a nominal *P*-value < 0.05 and false discovery rate (FDR) < 0.1 were considered significant. All statistical analyses were conducted using R software v4.3.1.

## Results

### Patients and treatments

Between March 2019 and February 2022, we enrolled 50 patients with HNSCC scheduled to receive definitive RT. Of these, seven patients with unavailable OS imaging data (six patients who did not undergo OS imaging and one patient whose OS imaging data could not be obtained due to a mechanical problem) were excluded. There were no patients who could not undergo OS imaging for the reason of invasiveness, indicating a minimally invasive nature of OS imaging. One patient who did not receive RT was also excluded from the analysis. Finally, 42 patients were analyzed for the primary and secondary endpoints (Fig. 1a).

All 42 patients completed RT. The median overall treatment time was 50 (range: 42–56) days. Forty-one (98%) patients received concurrent platinum-based chemotherapy, of whom 40 received cisplatin 80 mg/m^2^ once every 3 weeks, with a median cumulative dose of 240 (range: 80–240) mg/m^2^, and 1 patient received carboplatin (50–80 mg × l day/week) combined with 5-fluorouracil (250 mg × 2 days/week) due to renal dysfunction. The remaining patient did not receive chemotherapy due to old age.

### Oxygen saturation quantified by OS imaging

Of the 42 patients, the median pretreatment StO_2_ (61% [range: 45–87%]) was significantly lower in the tumors than in normal mucosa (66% [range: 54–84%], *P* = 0.004, Fig. 1b). The normality of pretreatment tumor StO₂ level distribution across patients, tested using the Shapiro–Wilk test, was *P* = 0.32, suggesting that the distribution was approximately normal (Fig. 1c). To stratify the cohort into two groups according to pretreatment StO₂ level, we used the cohort median (61%) as the cutoff value. We avoided selecting an outcome-driven optimal threshold (e.g., based on receiver operating characteristic analysis), as such an approach may introduce overfitting and increase the risk of incorrect findings, particularly in a relatively small dataset. As a result, of the 42 patients, 22 with tumor StO_2_ ≤ 61% were classified as the hypoxia group and 20 with tumor StO_2_ > 61% as the non-hypoxia group. The median pretreatment tumor StO_2_ levels in the hypoxia and non-hypoxia groups were 56.5% (range: 45–61%) and 69.5% (range: 62–87%), respectively. Fig. 1d, e show representative oxygen saturation images with pseudocolor maps of hypoxic- and non-hypoxic tumors and the distribution of StO_2_ in each type of tumor. The distributions of the pretreatment StO_2_ in each analyzed patient are shown in Supplementary Fig. 1a, b.

### Associations between pretreatment tumor StO_2_ and clinical outcomes

The baseline characteristics of the patients, including age, performance status, primary tumor site, tumor stage, nodal stage, disease stage, and chemotherapy regimen, were generally balanced between the hypoxia and non-hypoxia groups (Table 1). Among the 23 patients with oropharyngeal cancers, the proportion of patients with p16-positive tumors, who are thought to be more sensitive to RT^19^, was comparable between the hypoxia and non-hypoxia groups (83 vs. 100%).

**Table 1:** Baseline patient characteristics

Characteristic	Hypoxia group (*n* = 22)	Non-hypoxia group (*n* = 20)	P value
Age			
Median, years (range)	64 (46–77)	65 (45–80)	0.94
Sex			1.00
Male	18 (82%)	16 (80%)	
Female	4 (18%)	4 (20%)	
ECOG performance status			0.89
0	19 (86%)	16 (80%)	
1	3 (14%)	4 (20%)	
Primary tumor site			1.00
Oropharynx	12 (55%)	11 (55%)	
Hypopharynx	9 (41%)	8 (40%)	
Larynx	1 (4%)	1 (5%)	
p16 status in oropharyngeal cancer			0.50
Positive	10/12 (83%)	11/11 (100%)	
Negative	2/12 (17%)	0/11 (0%)	
Tumor stage^a^			0.28
T1	3 (14%)	3 (15%)	
T2	5 (23%)	7 (35%)	
T3	8 (36%)	7 (35%)	
T4	6 (27%)	3 (15%)	
Nodal stage^a^			0.48
N0	6 (27%)	2 (10%)	
N1	12 (55%)	11 (55%)	
N2	4 (18%)	6 (30%)	
N3	0 (0%)	1 (5%)	
Disease stage^a^			0.64
I	3 (14%)	3 (15%)	
II	7 (32%)	4 (20%)	
III	5 (23%)	8 (40%)	
IVA	6 (27%)	5 (25%)	
IVB	1 (4%)	0 (0%)	
Concurrent chemotherapy			0.37
Cisplatin	21 (96%)	19 (95%)	
Carboplatin+5-fluorouracil	1 (4%)	0 (0%)	
None	0 (0%)	1 (5%)	
Cumulative dose of cisplatin, mg/m^2^, median (range)	240 (80–240)^b^	240 (0–240)^c^	0.37

^a^Tumor, nodal and disease stages were assessed according to the 8th edition of Union for International Cancer Control stage classification system.

^b^One patient who received carboplatin and 5-fluorouracil was excluded from the hypoxia group.

^c^One patient in the non-hypoxia group did not receive any chemotherapy.

Abbreviation: ECOG=Eastern Cooperative Oncology Group.

We evaluated the response to RT at the primary tumor site, where the pretreatment tumor StO_2_ was measured. The primary endpoint, the CR rate of the primary tumor site at 8 weeks after RT completion, did not differ significantly between the hypoxia and non-hypoxia groups (86% [95% CI 65–97] vs. 100% [95% CI 83–100]; *P* = 0.23; Supplementary Fig. 2a). Three patients showed a non-CR, all of whom were in the hypoxia group. The median StO_2_ of the non-CR cases was 54% (range: 52–56%). In addition, we evaluated the CR rate of the primary tumor at 12 weeks after RT completion using cross-sectional imaging (CT, MRI, or PET) as a post hoc analysis. The CR rate did not differ significantly between the hypoxia and non-hypoxia groups (82% [95% CI 60–95] vs. 95% [95% CI 75–100], *P* = 0.35; Supplementary Fig. 2b). Among the patients classified as having a CR at 8 weeks after RT completion, one patient in each group (StO_2_ of 54% and 70%, respectively) progressed to non-CR at 12 weeks after RT completion (Supplementary Fig. 2c).

**Fig. 2:**
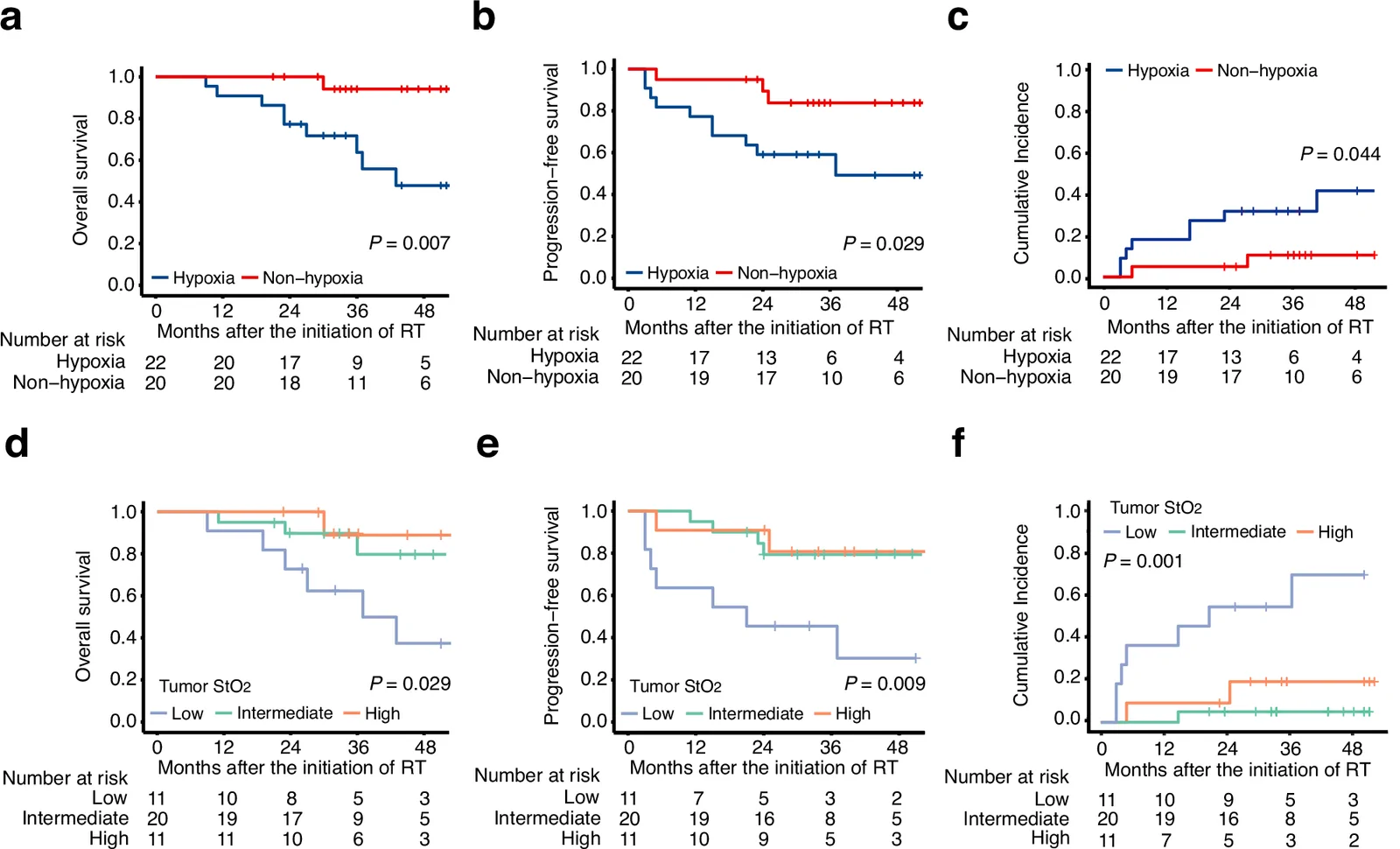
Clinical outcomes according to the pretreatment tumor StO_2_ quantified by OS imaging. **a**–**c** Patients with tumor StO_2_ ≤ 61% were assigned to the hypoxia group (*n* = 22) and those with tumor StO_2_ > 61% to the non-hypoxia group (*n* = 20). Overall survival (**a**), progression-free survival (**b**), and cumulative incidence of local failure (**c**) curves for the hypoxia and non-hypoxia groups. **d**–**f** Patients were stratified into three groups based on pretreatment StO₂ levels as follows: low StO₂ group, bottom 25th percentile (StO₂ ≤ 56%); intermediate StO₂ group, 25th to 75th percentile (StO_2_ level above 56% but 68% or below); high StO₂ group, ≥75th percentile (StO_2_ > 68%). Overall survival (**d**), progression-free survival (**e**), and cumulative incidence of local failure (**f**) curves for each of the three groups. *P* values were obtained by log-rank test (**a**–**f**).

The median follow-up duration was 36 (range: 9–53) months. Nine patients (41%) in the hypoxia group and one patient (5%) in the non-hypoxia group died during the follow-up period. The 1-year overall survival rate, a secondary endpoint, was 91% (95% CI 80–100) in the hypoxia group versus 100% (95% CI 100–100) in the non-hypoxia group (Fig. 2a). The 2-year overall survival rate was 77% (95% CI 62–97) in the hypoxia group versus 100% (95% CI 100–100) in the non-hypoxia group. The overall survival rate was significantly lower in the hypoxia than non-hypoxia group (*P* = 0.007).

Ten patients (45%) in the hypoxia group and three patients (15%) in the non-hypoxia group experienced disease progression during the follow-up period. The 1-year PFS rate was 77% (95% CI 62–97) in the hypoxia group versus 95% (95% CI 86–100) in the non-hypoxia group (Fig. 2b). The 2-year PFS rate was 59% (95% CI 42–84) in the hypoxia group versus 89% (95% CI 77–100) in the non-hypoxia group. The PFS rate was significantly lower in the hypoxia than non-hypoxia group (*P* = 0.029).

Eight patients (36%) in the hypoxia group and two (10%) in the non-hypoxia group experienced progression at the primary tumor site during the follow-up period. The 1-year cumulative incidence of LF at the primary tumor site was 18% (95% CI 5.5–37) in the hypoxia group versus 5% (95% CI 0.31–21) in the non-hypoxia group (Fig. 2c). The 2-year cumulative incidence of LF at the primary tumor site was 32% (95% CI 14–52) in the hypoxia group versus 5% (95% CI 0.31–21) in the non-hypoxia group. The LF rate was significantly higher in the hypoxia than non-hypoxia group (*P* = 0.044).

In a sensitivity analysis, patients were stratified into three groups based on pretreatment StO₂ levels, as follows: low StO₂ group, bottom 25th percentile (StO₂ ≤ 56%); intermediate StO₂ group, 25 to 75th percentile (StO_2_ level above 56% but 68% or below); high StO₂ group, ≥75th percentile (StO_2_ > 68%). The low StO₂ group exhibited poorer overall survival (*P* = 0.029; Fig. 2d), PFS (*P* = 0.009; Fig. 2e), and LF rates (*P* = 0.001; Fig. 2f) compared with the intermediate and high StO₂ groups.

### Relationship between tumor hypoxia quantified by OS imaging and gene expression-based hypoxia profile

**Fig. 3:**
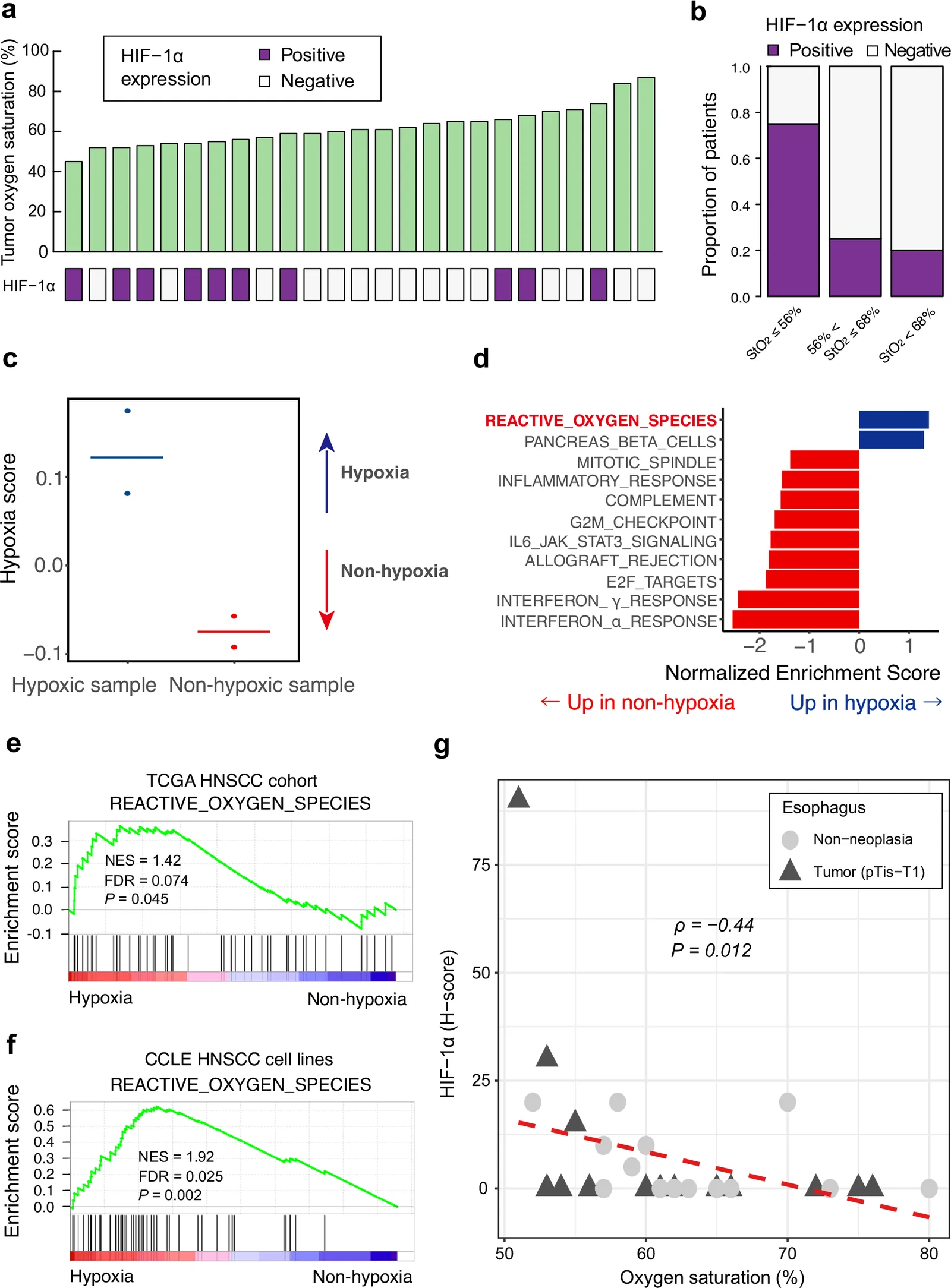
Tumor hypoxia quantified by OS imaging and gene expression-based hypoxia profile. **a** HIF-1α expression in HNSCC pretreatment biopsy specimens according to tumor StO_2_ levels (*n* = 25). **b** Proportion of HIF-1α-positivity based on StO₂ levels used to divide the cohort into the three groups (StO₂ ≤ 56%, *n* = 8; StO_2_ level > 56% and ≤ 68%, *n* = 12; StO_2_ > 68%, *n* = 5) shown in Fig. 2d–f. **c**,**d** RNA sequencing of hypoxic and non-hypoxic tumors. **c** Hypoxia scores of hypoxic (StO_2_: 59%, HIF-1α positive) and non-hypoxic (StO_2_: 70%, HIF-1α negative) tumor samples based on the HNSCC mRNA-based hypoxia signature. **d** Hallmark gene set enrichment analysis (GSEA) showing pathways upregulated in the hypoxic (blue) compared with non-hypoxic (red) tumor samples. **e**, **f** GSEA of HNSCC samples from the Cancer Genome Atlas (**e**) and Cancer Cell Lin**e** Encyclopedia (**f**) databases. Significant enrichment of the reactive oxygen species pathway in hypoxic compared with non-hypoxic samples (FDR and *P* value, see the Appendix: Mathematical Description of Methods of ref^16^.). FDR, false discovery rate; NES, normalized enrichment score. **g** Scatterplot showing the association of HIF-1α expression and StO_2_ levels in 32 esophageal specimens (16 superficial esophageal squamous cell carcinoma specimens and 16 non-neoplastic tissue specimens) from an independent cohort^13^ *P* and *ρ* values were calculated by Spearman’s rank correlation coefficient.

In the exploratory analysis, we evaluated the relationship between HIF-1α expression in pretreatment tumor biopsy specimens and StO_2_ levels. Of the 42 patients with HNSCC, we could assess HIF-1α expression in 25 patients for whom biopsy specimens were available. HIF-1α-positive HNSCC was enriched with lower StO₂ levels compared with HIF-1α-negative HNSCC (Fig. 3a). Specifically, when focusing on StO₂ levels used to divide the cohort into three groups (Fig. 2d–f), the low StO₂ group (StO₂ ≤ 56%) had a significantly higher proportion of HIF-1α-positive HNSCC than the intermediate and high StO₂ groups (*P* = 0.028: Fig. 3b).

Moreover, we performed RNA-seq of four fresh frozen tumor samples obtained from two patients before RT (two samples/patient). The median pretreatment StO_2_ of two tumors measured by OS imaging were 59% and 70%, and HIF-1α expression was positive and negative, respectively; the samples were defined as hypoxic and non-hypoxic samples, respectively. When focusing on expression-based hypoxia scores^14,15^, tumor StO_2_ measured by OS imaging reflected the expression-based hypoxia score (mean hypoxia score: 0.12 [range: 0.08–0.16] vs. −0.075 [range: −0.095 to −0.055] in the hypoxic vs. non-hypoxic samples; Fig. 3c). GSEA revealed that the reactive oxygen species (ROS) pathway was the most upregulated in the hypoxic samples compared with the non-hypoxic samples (*P* < 0.05 and FDR < 0.1; Fig. 3d), consistent with the generation of ROS during hypoxic stress by mitochondrial complex III^20^. GSEA of 560 HNSCC cases from the TCGA dataset and 60 HNSCC cell lines from the CCLE dataset confirmed that the ROS pathway was significantly upregulated in the hypoxic samples compared with the non-hypoxic samples (*P* < 0.05 and FDR < 0.1; Fig. 3e, f).

Finally, we analyzed an independent cohort of 32 esophageal specimens (16 superficial esophageal squamous cell carcinoma specimens and 16 non-neoplastic tissue specimens) to validate the relationship between StO₂ and HIF-1α expression levels. StO₂ values were significantly inversely correlated with HIF-1α expression levels (Spearman’s *ρ* = −0.44, *P* = 0.012; Fig. 3g). Overall, these findings suggest a biological association between reduced StO_2_ levels measured by OS imaging and hypoxia-related molecular activation.

## Discussion

In this prospective observational study, we evaluated the association between pretreatment tumor StO_2_ quantified by OS imaging and the clinical outcomes of patients with HNSCC receiving RT, predominantly with concurrent chemotherapy. This study did not meet its primary endpoint; the CR rate of the primary tumor site at 8 weeks after RT completion did not differ significantly between the hypoxia and non-hypoxia groups. However, the hypoxia group had lower 1-year overall survival and PFS rates and a higher LF rate compared with the non-hypoxia group. Our findings suggest that OS imaging may potentially identify hypoxic tumors with resistance to RT in HNSCC.

In previous studies of RT for HNSCC, poor clinical outcomes have been reported in patients with hypoxic tumors determined by PET/CT using the nitroimidazole-based, hypoxia-specific tracers ^18^F-fluoroazomycin arabinoside and ^18^F-fluoromisonidazole^21,22^. However, nitroimidazole-based hypoxia imaging requires specialized radiopharmaceutical production and dedicated PET/CT facilities. In addition, these radiopharmaceuticals are not covered by national health insurance in Japan; therefore, we cannot use hypoxia-targeting radiopharmaceuticals in clinical practice. Although PET/CT with ^18^F-fluorodeoxyglucose can potentially detect hypoxic tumors and is widely used in oncology^23^, its ability to detect hypoxia is limited because it is not a hypoxia-specific tracer^24^. Consequently, availability in routine clinical practice remains limited. On the other hand, OS imaging can easily assess tumor hypoxia in addition to conventional endoscopic images without the need for additional invasive procedures. Therefore, OS imaging has the potential to be widely used in clinical practice. Also, our study supports the accuracy of OS imaging in identifying tumor hypoxia. The median StO_2_ quantified by OS imaging was significantly lower in the tumor than normal mucosa, consistent with our previous studies in the esophagus, stomach, and colon^11,13^.

The strength of our study is that we prospectively evaluated the associations between pretreatment tumor StO_2_ and clinical outcomes. To our knowledge, no prospective studies have investigated the impact of tissue oxygenation levels measured by endoscopy on clinical outcomes for any tumor type. We hypothesize that the limited number of LF events in our cohort contributed to the lack of a difference in the CR rate between the hypoxia and non-hypoxia groups. On the other hand, the hypoxia group had worse 1-year overall survival, PFS, and LF rates compared with the non-hypoxia group. For patients with locally advanced HNSCC in whom both surgical resection and definitive RT/chemoradiotherapy are indicated, assessment of tumor hypoxia levels may help guide therapeutic decisions. For radiation oncologists, identifying hypoxic HNSCC before treatment could provide a rationale for increasing the treatment intensity, such as dose escalation or the use of radiosensitizers. Furthermore, the minimally invasive and easily repeatable nature of OS imaging may be suitable for evaluating temporal and spatial changes in tumor hypoxic regions during RT.

Our study has several potential limitations. First, the cut-off value for stratifying our cohort into the hypoxia and non-hypoxia groups cannot be generalized because the number of patients in our cohort was small. However, to eliminate arbitrariness, we applied the median StO_2_ level as the cut-off value. We have already started a prospective study (UMIN000057661) with a larger number of patients with HNSCC to explore a more accurate cut-off StO_2_ value for detecting radioresistant tumors. Second, the biological assessments of the ability of OS imaging to detect tumor hypoxia were insufficient. We assessed the association between HIF-1α expression in tumors pretreatment and tumor StO_2_ in approximately 60% of patients. RNA-seq was performed using only one hypoxic and one non-hypoxic tumor detected by OS imaging. However, our results were validated using external datasets. In our aforementioned prospective study, we will collect tumor samples from all patients simultaneously undergoing OS imaging for biological evaluation. Third, the cohort size was small, which may limit the statistical power. However, the observed effect sizes were clinically meaningful. The absolute differences at 1 year were 9% for overall survival, 18% for PFS, and 13% for LF. The consistency of the effect across analyses further supports the potential clinical relevance of our findings. Fourth, due to limited sample size, only univariate analyses were performed to evaluate the association between StO₂ levels and survival outcomes. Therefore, the independent prognostic value of StO₂ could not be assessed fully in a multivariable model. In our aforementioned prospective study with a larger cohort, the independent value of StO₂ should be addressed. Fifth, we evaluated human papillomavirus (HPV) status using p16 immunohistochemistry, which may not fully reflect transcriptionally active HPV infection^25^. Furthermore, the number of HPV-negative HNSCCs in our cohort was limited (n = 2), making subgroup analysis difficult. Therefore, the potential impact of HPV status on the association between tumor hypoxia and clinical outcomes should be interpreted with caution. HPV-positive oropharyngeal cancer responds well to RT/chemoradiotherapy (CRT); therefore, RT/CRT is often recommended. In contrast, since RT/CRT is generally less effective for HPV-negative HNSCC, we believe it is important to find factors to help guide therapeutic decisions for patients with HPV-negative HNSCC eligible for surgical resection and definitive radiotherapy. For this reason, this study and the aforementioned ongoing prospective study include patients with HPV-negative HNSCC eligible for surgical resection and definitive radiotherapy. Finally, OS imaging measures the oxygenation status of the tumor mucosa and may not fully reflect that of the entire tumor. However, previous studies have suggested that superficial tumor oxygenation assessed by near-infrared spectroscopy may correlate with intratumoral oxygenation measured using a needle electrode^26^ and pimonidazole-defined hypoxia^27^. Furthermore, hypoxia-related biomarkers are often evaluated using biopsy specimens obtained from the tumor mucosa.

In conclusion, the pretreatment tumor StO_2_ quantified by OS imaging was correlated with the 1-year overall survival, PFS, and LF rates after RT. Our findings suggest that OS imaging can potentially be used to identify hypoxic tumors with poor outcomes after RT, and they warrant further prospective studies to validate the impact of OS imaging on clinical outcomes and to determine whether OS imaging can predict the RT response in HNSCC.

## Supplementary information

Supplemental Information

Description of Additional Supplementary files

Supplementary Data

## Data Availability

The patient-specific data generated in this study, including the transcriptome data that support the study findings, are not publicly available due to patient privacy requirements and ethical restrictions defined in the informed consent document. Data are available upon reasonable request from the corresponding author (H.H.) and subject to approval by the IRB. An initial response to data access requests will be provided within 15 business days of receipt. Access to patient-specific data is restricted to qualified researchers who have received IRB approval and to the approved research purpose. Any request will be reviewed to determine whether the request is subject to any intellectual property or confidentiality obligations. All data shared will be de-identified. Source data not subject to these restrictions (Figs. 1 and 3) are available within their supplementary data files. RNA-seq data from patients with HNSCC in the TCGA dataset are available on the National Cancer Institute’s Genomic Data Commons (HNSC_RNASeqV2_final_geneExp, https://gdc.cancer.gov/about-data/publications/PanCan-Squamous-2018/). RNA-seq data from HNSCC cell lines in the CCLE dataset are available on the Cancer Dependency Map portal (Expression Public 23Q2, https://depmap.org/portal/).

## References

[1] Sung, H. et al. Global Cancer Statistics 2020: GLOBOCAN estimates of incidence and mortality worldwide for 36 cancers in 185 countries. *CA Cancer J. Clin.* **71**, 209–249 (2021). 10.3322/caac.21660 33538338

[2] Grégoire, V., Langendijk, J. A. & Nuyts, S. Advances in radiotherapy for head and neck cancer. *J. Clin. Oncol.* **33**, 3277–3284 (2015). 10.1200/JCO.2015.61.2994 26351354

[3] Johnson, D. E. et al. Head and neck squamous cell carcinoma. *Nat. Rev. Dis. Prim.* **6**, 92 (2020). 10.1038/s41572-020-00224-3 PMC7944998 33243986

[4] Linge, A. et al. Low cancer stem cell marker expression and low hypoxia identify good prognosis subgroups in HPV(-) HNSCC after postoperative radiochemotherapy: a multicenter study of the DKTK-ROG. *Clin. Cancer Res.* **22**, 2639–2649 (2016). 10.1158/1078-0432.CCR-15-1990 26755529

[5] Göttgens, E. L., Ostheimer, C., Span, P. N., Bussink, J. & Hammond, E. M. HPV, hypoxia and radiation response in head and neck cancer. *Br. J. Radiol.* **92**, 20180047 (2019). 10.1259/bjr.20180047 PMC6435089 29493265

[6] Nordsmark, M. et al. Prognostic value of tumor oxygenation in 397 head and neck tumors after primary radiation therapy. An international multi-center study. *Radiother. Oncol.* **77**, 18–24 (2005). 10.1016/j.radonc.2005.06.038 16098619

[7] Bhandari, V., Li, C. H., Bristow, R. G. & Boutros, P. C. Divergent mutational processes distinguish hypoxic and normoxic tumours. *Nat. Commun.* **11**, 737 (2020). 10.1038/s41467-019-14052-x PMC7002770 32024819

[8] Horsman, M. R., Mortensen, L. S., Petersen, J. B., Busk, M. &Overgaard, J. Imaging hypoxia to improve radiotherapy outcome. *Nat. Rev. Clin. Oncol.* **9**, 674–687 (2012). 10.1038/nrclinonc.2012.171 23149893

[9] Vaupel, P., Höckel, M. & Mayer, A. Detection and characterization of tumor hypoxia using pO2 histography. *Antioxid. Redox Signal.* **9**, 1221–1235 (2007). 10.1089/ars.2007.1628 17536958

[10] Grimes, D. R., Warren, D. R. & Warren, S. Hypoxia imaging and radiotherapy: bridging the resolution gap. *Br. J. Radiol.* **90**, 20160939 (2017). 10.1259/bjr.20160939 PMC5603947 28540739

[11] Kaneko, K. et al. Hypoxia imaging endoscopy equipped with laser light source from preclinical live animal study to first-in-human subject research. *PLoS One* **9**, e99055 (2014). 10.1371/journal.pone.0099055 PMC4051687 24915532

[12] Koganemaru, S. et al. Quantitative analysis of the concentration of trifluridine in tumor hypoxic regions using a novel platform combining functional endoscopy and mass spectrometry. *Clin. Pharmacol. Ther.* **115**, 62–70 (2024). 10.1002/cpt.3066 37803526

[13] Minakata, N. et al. Immunohistochemistry and oxygen saturation endoscopic imaging reveal hypoxia in submucosal invasive esophageal squamous cell carcinoma. *Cancer Med.* **12**, 15809–15819 (2023). 10.1002/cam4.6217 PMC10469640 37329213

[14] Ye, Y. et al. Characterization of hypoxia-associated molecular features to aid hypoxia-targeted therapy. *Nat. Metab.* **1**, 431–444 (2019). 10.1038/s42255-019-0045-8 PMC6980239 31984309

[15] Winter, S. C. et al. Relation of a hypoxia metagene derived from head and neck cancer to prognosis of multiple cancers. *Cancer Res.* **67**, 3441–3449 (2007). 10.1158/0008-5472.CAN-06-3322 17409455

[16] Subramanian, A. et al. Gene set enrichment analysis: a knowledge-based approach for interpreting genome-wide expression profiles. *Proc. Natl. Acad. Sci. USA* **102**, 15545–15550 (2005). 10.1073/pnas.0506580102 PMC1239896 16199517

[17] Campbell, J. D. et al. Genomic, pathway network, and immunologic features distinguishing squamous carcinomas. *Cell Rep.* **23**, 194–212.e6 (2018). 10.1016/j.celrep.2018.03.063 PMC6002769 29617660

[18] Hirata, H. et al. The evolving genomic landscape of esophageal squamous cell carcinoma under chemoradiotherapy. *Cancer Res.* **81**, 4926–4938 (2021). 10.1158/0008-5472.CAN-21-0653 34413060

[19] Ang, K. K. et al. Human papillomavirus and survival of patients with oropharyngeal cancer. *N. Engl. J. Med.* **363**, 24–35 (2010). 10.1056/NEJMoa0912217 PMC2943767 20530316

[20] Lee, P., Chandel, N. S. & Simon, M. C. Cellular adaptation to hypoxia through hypoxia inducible factors and beyond. *Nat. Rev. Mol. Cell Biol.* **21**, 268–283 (2020). 10.1038/s41580-020-0227-y PMC7222024 32144406

[21] Saksø, M. et al. Influence of FAZA PET hypoxia and HPV-status for the outcome of head and neck squamous cell carcinoma (HNSCC) treated with radiotherapy: Long-term results from the DAHANCA 24 trial (NCT01017224). *Radiother. Oncol.* **151**, 126–133 (2020). 10.1016/j.radonc.2020.08.006 32805273

[22] Welz, S. et al. Dose escalation to hypoxic subvolumes in head and neck cancer: a randomized phase II study using dynamic [^18^F]FMISO PET/CT. *Radiother. Oncol.* **171**, 30–36 (2022). 10.1016/j.radonc.2022.03.021 35395276

[23] Tamaki, N. et al. Four-dimensional quantitative analysis using FDG-PET in clinical oncology. *Jpn. J. Radiol.* **41**, 831–842 (2023). 10.1007/s11604-023-01411-4 PMC10366296 36947283

[24] Nehmeh, S. A. et al. Comparison of FDG and FMISO uptakes and distributions in head and neck squamous cell cancer tumors. *EJNMMI Res.* **11**, 38 (2021). 10.1186/s13550-021-00767-w PMC8046891 33855685

[25] Mehanna, H. et al. Prognostic implications of p16 and HPV discordance in oropharyngeal cancer (HNCIG-EPIC-OPC): a multicentre, multinational, individual patient data analysis. *Lancet Oncol.* **24**, 239–251 (2023). 10.1016/S1470-2045(23)00013-X 36796393

[26] Kim, J. G. et al. Interplay of tumor vascular oxygenation and tumor pO₂ observed using near-infrared spectroscopy, an oxygen needle electrode, and ¹⁹F MR pO₂ mapping. *J. Biomed. Opt.* **8**, 53–62 (2003). 10.1117/1.1527049 12542380

[27] Zhao, D. et al. In vivo near-infrared spectroscopy and magnetic resonance imaging monitoring of tumor response to combretastatin A-4-phosphate correlated with therapeutic outcome. *Int. J. Radiat. Oncol. Biol. Phys.* **80**, 574–581 (2011). 10.1016/j.ijrobp.2010.12.028 PMC3090506 21345614

