# Supplementary material for: Prospective evaluation of oxygen saturation endoscopic imaging for radiotherapy response in head and neck cancer
Source: Commun Med (Lond). 2026 Jul 1;6:553. doi: 10.1038/s43856-026-01752-x (PMC13691073; doi:10.1038/s43856-026-01752-x)
Supplement: Supplementary file 1 [file 43856_2026_1752_MOESM1_ESM.pdf]

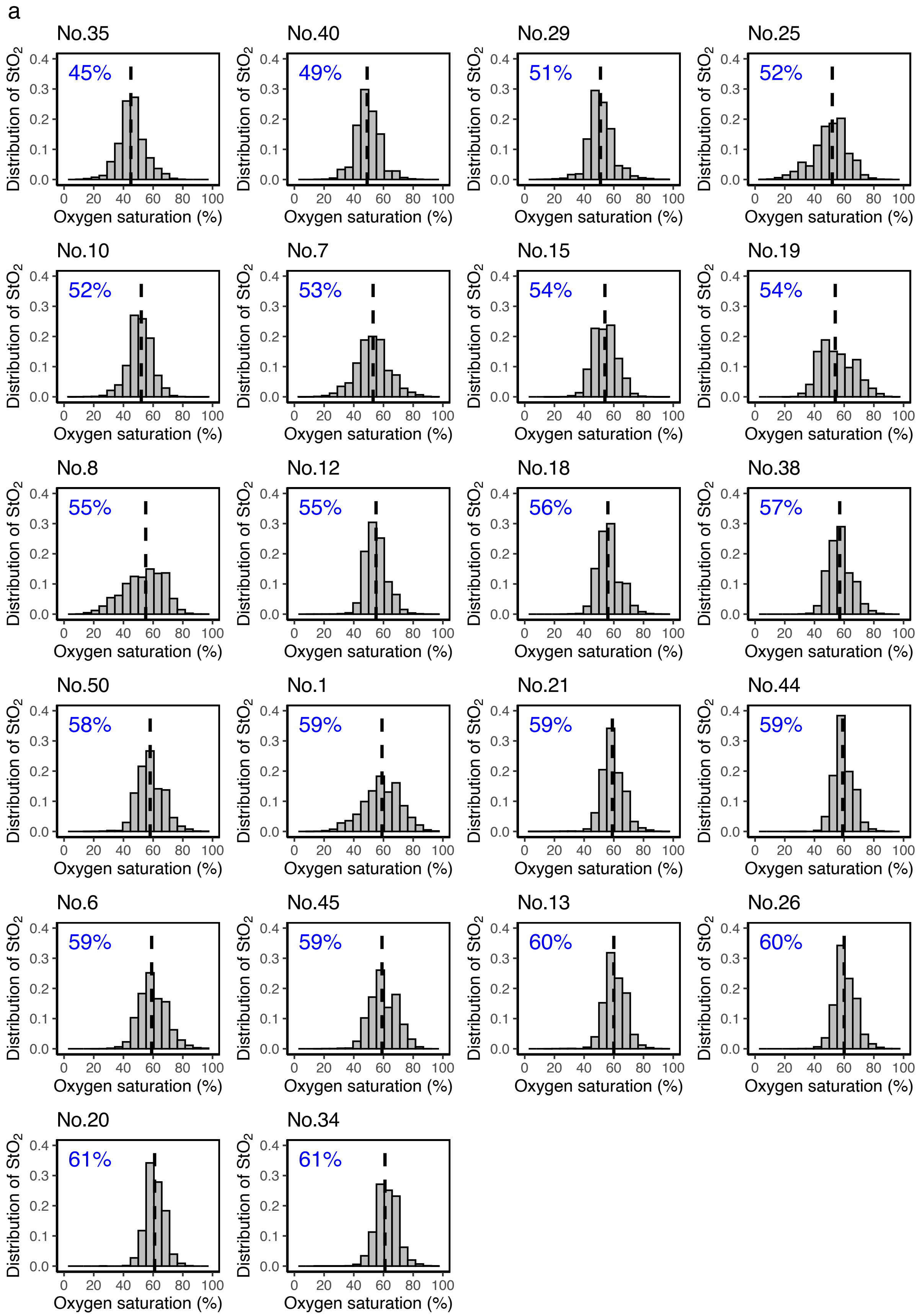
a
No.35
45%
No.40
49%
No.29
51%
No.25
52%
No.10
52%
No.7
53%
No.15
54%
No.19
54%
No.8
55%
No.12
55%
No.18
56%
No.38
57%
No.50
58%
No.1
59%
No.21
59%
No.44
59%
No.6
59%
No.45
59%
No.13
60%
No.26
60%
No.20
61%
No.34
61%
Distribution of StO2
Oxygen saturation (%)
0 20 40 60 80 100
0.0 0.1 0.2 0.3 0.4

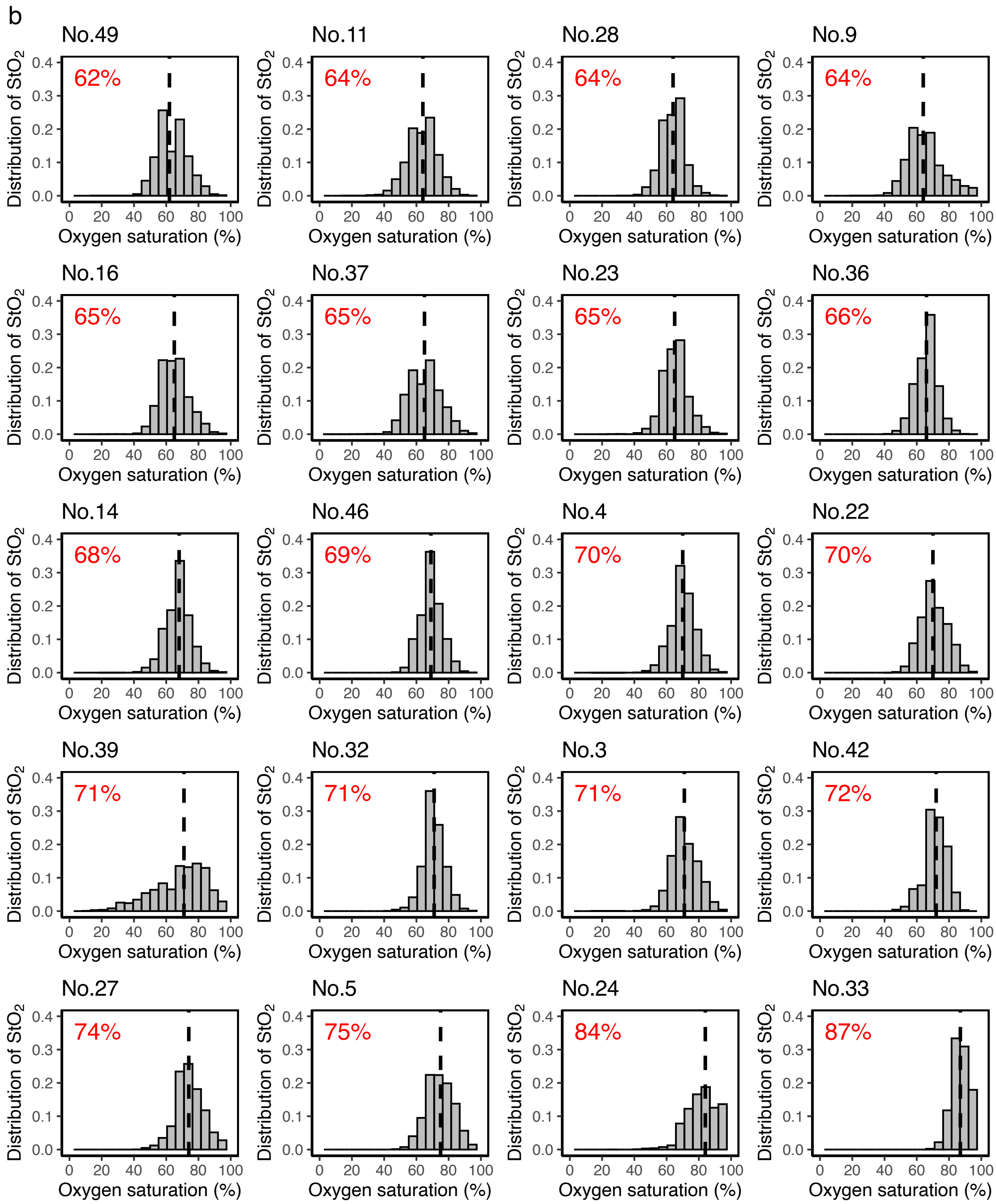

Supplementary Figure 1. Histograms representing the distribution of pretreatment tumor StO_2 levels measured by OS imaging in each patient in the hypoxia ($StO_2 \leq 61\%$, n = 22) and non-hypoxia ($StO_2 > 61\%$, n = 20) groups. **a).** Hypoxia group. **b).** Non-hypoxia group.

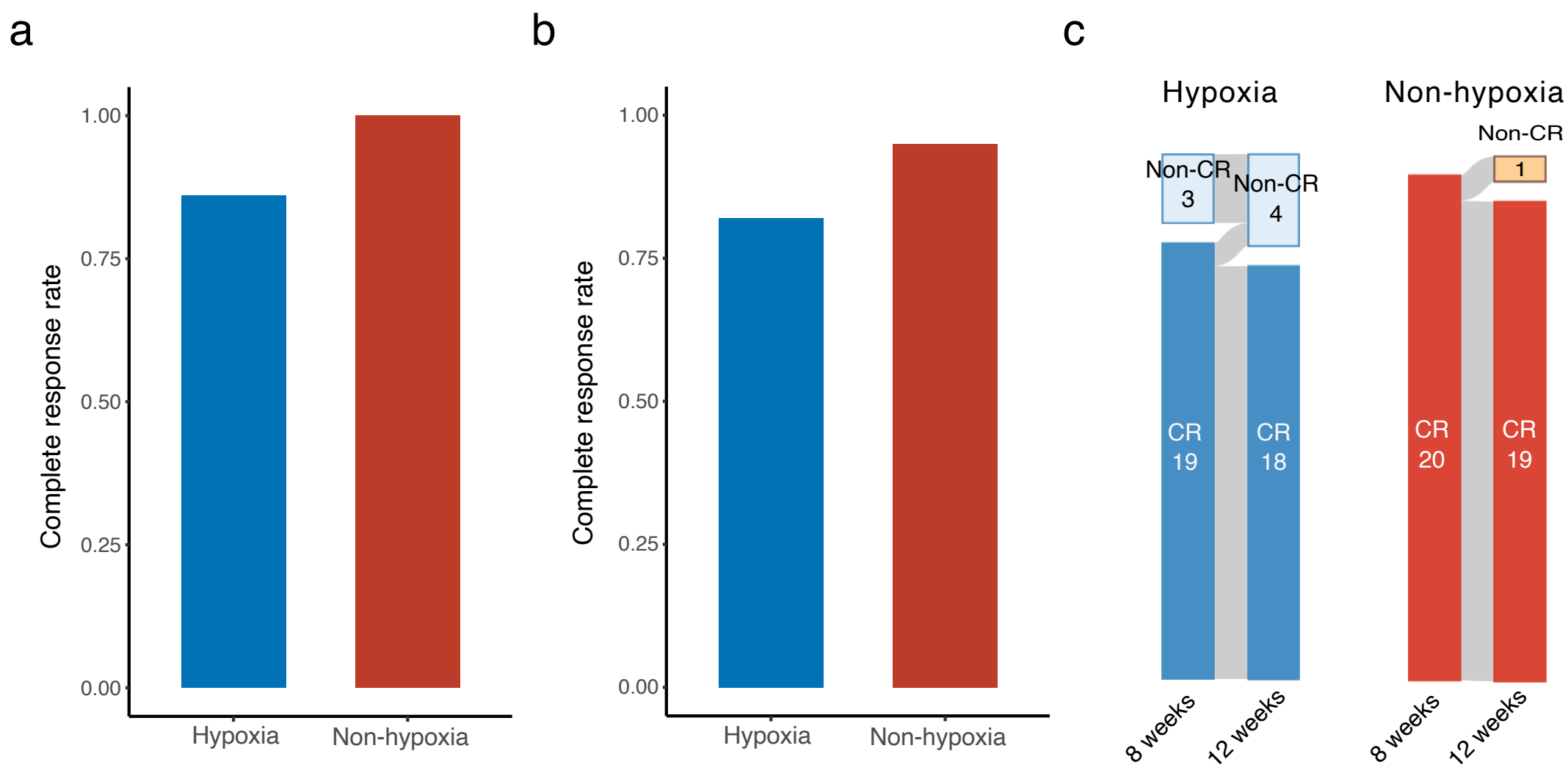

Supplementary Figure 2. Complete response (CR) rates at 8 weeks (**a**) and 12 weeks (**b**) after RT completion in the hypoxia and non-hypoxia groups. **c).** Changes in the number of patients showing a CR and non-CR from 8 weeks to 12 weeks after RT completion in each group.
