# Supplementary material for: Prospective evaluation of oxygen saturation endoscopic imaging for radiotherapy response in head and neck cancer
Source: Commun Med (Lond). 2026 Jul 1;6:553. doi: 10.1038/s43856-026-01752-x (PMC13691073; doi:10.1038/s43856-026-01752-x)
Supplement: Supplementary file 2 [file 43856_2026_1752_MOESM2_ESM.docx]

**Description of Additional Supplementary Files**

File name: Supplementary Data

Description: Source data for figure 1 & 3
